# Cell tracking *in vitro* reveals that the extracellular matrix glycoprotein Tenascin-C modulates cell cycle length and differentiation in neural stem/progenitor cells of the developing mouse spinal cord

**DOI:** 10.1242/bio.027730

**Published:** 2018-07-15

**Authors:** Marcus May, Bernd Denecke, Timm Schroeder, Magdalena Götz, Andreas Faissner

**Affiliations:** 1 Department for Cell Morphology and Molecular Neurobiology, Ruhr-University Bochum, 44780 Bochum, Germany; 2 Aachen Interdisciplinary Center for Clinical Research, Faculty of Medicine, Rheinisch-Westfälische Technische Hochschule Aachen, 52074 Aachen, Germany; 3 Department of Biosystems Science and Engineering, ETH Zürich, 4058 Basel, Switzerland; 4 Physiological Genomics, Biomedical Center, Ludwig-Maximilians University Munich, 82152 Planegg/Martinsried, Germany; 5 Department of Physiological Genomics, Biomedical Center, Ludwig-Maximilians University Munich, 82152 Planegg/Martinsried, Germany; 6 Munich Cluster for Systems Neurology (SyNergy), Ludwig-Maximilians University Munich, 81377 Munich, Germany

**Keywords:** Extracellular matrix, Cell lineage, Gliogenesis, Growth factor responsiveness, Stem cell niche, Tenascin-C, Time-lapse video microscopy

## Abstract

Generation of astrocytes during the development of the mammalian spinal cord is poorly understood. Previously, we have shown that the glycoprotein of the extracellular matrix (ECM) tenascin-C (Tnc) modulates the expression territories of the patterning genes Nkx6.1 and Nkx2.2 in the developing ventral spinal cord, tunes the responsiveness of neural stem/progenitor cells towards the cytokines FGF2 and EGF and thereby promotes astrocyte maturation. In order to obtain further mechanistic insight into these processes, we have compared embryonic day-15 spinal cord neural progenitor cells (NPCs) from wild-type and *Tnc* knockout mice using continuous single-cell live imaging and cell lineage analysis *in vitro*. *Tnc* knockout cells displayed a significantly reduced rate of cell division both in response to FGF2 and EGF. When individual clones of dividing cells were investigated with regard to their cell lineage trees using the tTt tracking software, it appeared that the cell cycle length in response to growth factors was reduced in the knockout. Furthermore, when *Tnc* knockout NPCs were induced to differentiate by the removal of FGF2 and EGF glial differentiation was enhanced. We conclude that the constituent of the stem cell niche Tnc contributes to preserve stemness of NPCs.

## Abstract (precis)

**Summary:** We have shown, using digital video microscopy based cell lineage tracing, that the glycoprotein of the extracellular matrix tenascin-C modulates growth factor responsiveness and cell cycle length of embryonic spinal cord progenitor cells *in vitro*.

## INTRODUCTION

The extracellular matrix (ECM) is a highly dynamic structure that modulates cell proliferation, migration and differentiation processes in the healthy and diseased central nervous system (CNS) (Barros et al., 2011; Bonnans et al., 2014; Wiese and Faissner, 2015). Collagens, proteoglycans and glycoproteins are the main components of the ECM, creating a tissue specific spatio-temporal microenvironment (Faissner and Reinhard, 2015; Rauch, 2007).

Tnc is a prominent glycoprotein of the ECM in the CNS and consists of four distinct domains: a cysteine-rich assembly domain, 14.5 epidermal growth factor-like repeats, a series of fibronectin type III-like (FNIII) repeats and a C-terminal fibrinogen-like globe (Chiquet-Ehrismann and Tucker, 2011; Joester and Faissner, 2001). During embryonic development Tnc is widely expressed, downregulated thereafter and persists in the stem cell niches of several organs, including the CNS (Chiquet-Ehrismann et al., 2014). In the CNS, Tnc is synthesized by the radial glia stem cells of the ventricular zone, in the olfactory bulb (Bartsch et al., 1992; Gates et al., 1995; Götz et al., 1997; Treloar et al., 2009), and by oligodendrocyte and astrocyte precursor cells (Czopka et al., 2009; Garcion et al., 2001; Garwood et al., 2004; Prieto et al., 1990; Tucker et al., 1994). In stem and glial progenitor cells (NPCs), Tnc influences their self-renewal, maintenance and differentiation by modulating growth factor responsiveness towards the FGF2- and EGF-dependent signalling pathways, and regulating the expression of the guanine nucleotide exchange factor Vav3 and the RNA-binding protein Sam68 (Czopka et al., 2010; Faissner et al., 2017; Garcion et al., 2004; Moritz et al., 2008). There is evidence that *Tnc* is controlled by the paired-box transcription factor 6 (Pax6), because transient overexpression of Pax6 in neurospheres resulted in the up-regulation of Tnc isoforms containing four to six alternatively spliced FNIII repeats (von Holst et al., 2007). Conversely, Tnc expression is modified in the natural Pax6 mutant small eye (*sey*) (Götz et al., 1998; Karus et al., 2011). Pax6 regulates patterning, neurogenesis and proliferation in forebrain development, which requires an intact DNA binding domain (Walcher et al., 2013). Interestingly, the overexpression of Pax6 in the embryonic mouse brain generates more basal progenitors (BP) (Asami et al., 2011; Wong et al., 2015). Furthermore, Tnc is also expressed by human outer radial glia cells and is presumably involved in human cortical development (Pollen et al., 2015). With ongoing maturation Tnc becomes downregulated and is restricted to the adult neural stem cell niches, the subventricular zone at the lateral wall of the lateral ventricle, the rostral migratory stream and the subgranular zone of the hippocampus (Kazanis et al., 2007; Kempermann et al., 2004; Miragall et al., 1990). Tnc is strongly upregulated in reactive astrocytes upon lesion and in a broad range of carcinomas (Midwood and Orend, 2009; Roll et al., 2012; Wiese et al., 2012).

In the embryonic spinal cord, Tnc expression occurs around the central canal at E13.5, extends to the ventral part around E15 and is generalized in the spinal cord at E18. The genetic ablation of Tnc leads to reduced FGF2 signalling and delayed maturation of astrocyte progenitors (Karus et al., 2011). In the adult spinal cord, Tnc expression is associated with motoneurons and ependymal cells (Zhang et al., 1995).

In summary, compelling evidence suggests a link between Tnc expression and the proliferation and differentiation of NPCs. However, it is not possible to follow the lineage relationships of NPCs in the embryonic mouse spinal cord *in situ.* In order to analyse the effect of Tnc on EGF- and FGF2-related signalling in murine spinal cord progenitors on the cellular level, we performed time lapse-video microscopy and single-cell tracking *in vitro* to generate lineage trees and to obtain information concerning the cell division mode (Costa et al., 2011; Eilken et al., 2009; Hoppe et al., 2016; Rieger and Schroeder, 2009). Here we show that in the absence of Tnc the mitotic response of NPCs to the growth factors FGF2 and EGF is strongly reduced. Within the subpopulation of dividing cells, FGF2 exposure leads to a shorter cell cycle in comparison with EGF treatment in both wild-type (WT) and Tnc knockout (KO) progenitors. In addition, cells treated with EGF and FGF2 divided faster in the absence of Tnc. To our knowledge, this is the first report that the glycoprotein Tnc of the ECM has an impact on the cell cycle length of spinal cord progenitors.

## RESULTS

### Time-lapse video microscopy reveals a diminished mitotic rate of Tnc KO spinal cord progenitor cells

In order to study the impact of the glycoprotein Tnc of the ECM on the cell biology of neural stem cells, we examined E15 spinal cord progenitor cells by time-lapse video microscopy in culture. First, the adequate conditions of the cell culture substrate were examined. When wild-type radial glia stem cells were cultivated on poly-D-Lysine coated with mouse CNS-derived Tnc, the cells detached and either formed aggregates or evaded into the culture medium (data not shown). This mirrors the anti-adhesive properties of Tnc that had been reported for CNS neurons (Faissner and Kruse, 1990; Joester and Faissner, 2001). It appeared that the cultures developed most successfully on a substrate composed of poly-D-lysine (PDL) in conjunction with laminin-1 that is also used for differentiation assays of neurospheres (von Holst et al., 2007). Because Tnc substrates could not be investigated, we chose to compare stem cells from wild-type and Tnc KO mice to gain insight into the functions of this extracellular matrix glycoprotein in the stem cell compartment (Faissner et al., 2017). Initially, we used E15 WT and Tnc KO spinal cord progenitors in the absence of the cytokines FGF2 and EGF. Under these conditions, however, only a few dividing cells were visible. Some developed differentiated glial morphologies while the majority of cells eventually vanished, resulting in an overall shrinking population (see Movie 1). This reflects the low survival rates of embryonic spinal cord radial glia stem cells deprived of growth factors.

**Fig. 1.**
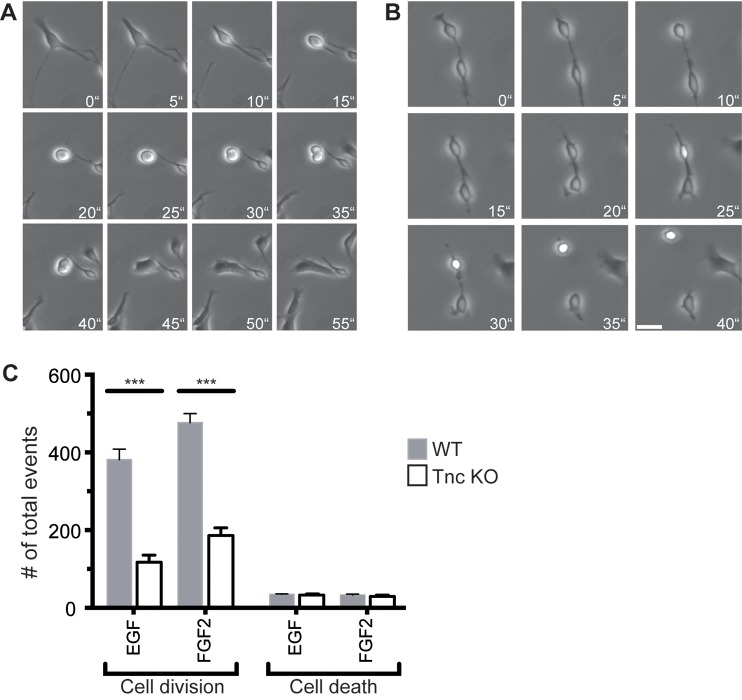
**Tnc deficiency resulted in reduced cell division both in response to EGF and FGF2.** (A) Phase contrast images of time-lapse video microscopy of a typical cell division are shown. After 30 min, two daughter cells are visible for the first time. (B) An example of a dying cell is illustrated. The cell rounded up, became bright and finally died. (C) Quantification of the total number of both cell divisions and cell deaths revealed a massive reduction of cell divisions in Tnc-deficient cell populations in comparison to the WT, independently from the EGF or FGF2 treatment. Cell death is not affected. Error bars indicate s.d. ***P≤0.001 (*t*-test); *n*=4; Scale bar: 50 µm.

Therefore, we maintained progenitor cells in the presence of EGF and FGF2 and determined the total number of cell divisions and cell deaths over 2.5 days by counting every single-cell division and each dying cell in phase contrast images obtained by time-lapse video microscopy. A typical cell division and a dying cell are depicted as an example for both events (Fig. 1A,B). The quantification displayed an intense reduction in cell divisions of progenitors lacking Tnc in comparison with WT cells in both the EGF and the FGF2 condition (Fig. 1C). The total number of dividing cells was decreased by about 70% and 60% in the presence of EGF and FGF2, respectively (EGF: WT, 382±54; Tnc KO, 118±23. FGF2: WT, 477±57; Tnc KO, 187±33; *n*=4; *P*≤0.001) (Fig. 1C). In contrast to this observation, the number of dying cells was similar in WT and Tnc KO progenitors (EGF: WT, 35±2; Tnc KO, 33±6. FGF2: WT, 33±6; Tnc KO, 29±10) (Fig. 1C). In summary, Tnc KO progenitors divided less often in comparison with WT cells, but cell death was not affected.

### Time-lapse video microscopy elucidates cell cycle progression of spinal cord progenitors

**Fig. 2.**
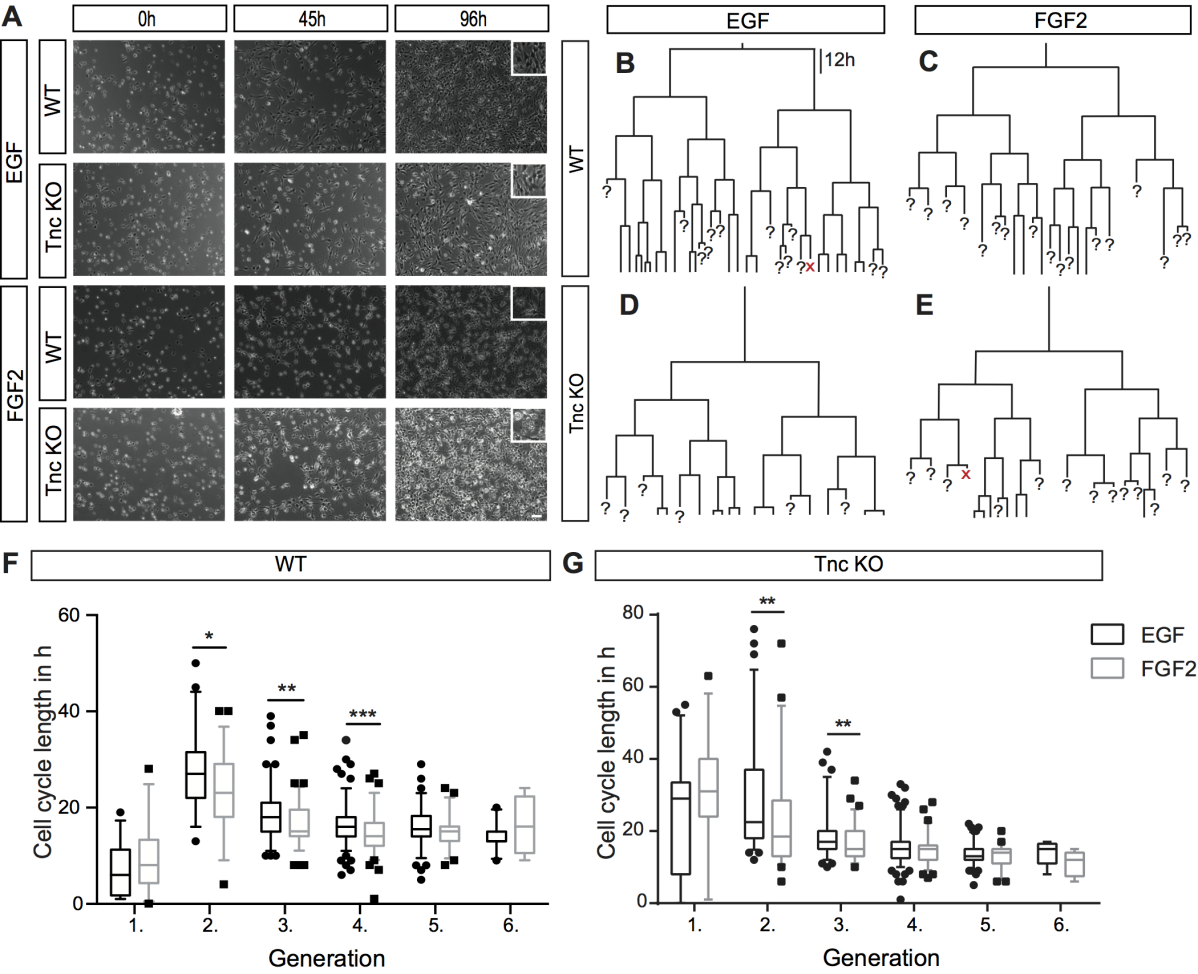
**Cell cycle length of WT and Tnc KO spinal cord progenitors is decreased upon FGF2- in comparison to EGF-treatment.** (A) Phase contrast images sustained by time-lapse video microscopy at 0 h, 45 h and 96 h of WT and Tnc KO progenitors cultivated in presence of EGF or FGF2. After 96 h, the substrate was covered with cell monolayers under all conditions. Note that FGF2-treatment led to a bipolar cell morphology; in contrast EGF-treated cells displayed a larger cell body. (B-E) Typical lineage trees of WT and Tnc-deficient progenitors tracked in the presence of EGF or FGF2. It was possible to follow a minority of cells until their sixth division, which is the sixth generation. Most sibling-cells divided synchronously within a time span of a few hours difference. Question marks indicates that the cell was not traceable any further and ‘x’ represents a dying cell. (D,E) The number of branching points in the lineage tree is reduced in the KO (18 versus 35 in the presence of EGF; B,D), reflecting the smaller number of cell divisions recorded in the traced pedigree. (F,G) The cell cycle length of WT and Tnc KO spinal cord progenitors with regard to EGF and FGF2 is shown. (F,G) Filled circles, EGF condition; filled squares, FGF2 condition; individual values beyond the 5%-95% range of the box whisker plot. There is reduction in cell cycle length with rising generation under EGF and FGF2 conditions. Furthermore, FGF2 drove progenitors to divide faster in the second to fourth generation in comparison to EGF. The same tendencies can be documented in Tnc KO spinal cord progenitors. Note that in the second and third generations, the treatment with FGF2 led to faster division of progenitors (G). *P≤0.05, **P≤0.01, ***P≤0.001 (Mann-Whitney *U*-test); *n*=4; percentile: 5%­–95%; scale bar: 50 µm.

In order to have a closer look at the dividing subpopulation of the progenitors we performed single-cell tracking; we focused our attention on the subpopulation of individual dividing cells. To this end, an individual cell in a time-lapse video microscopy recording was marked and the progeny followed for up to six generations using the tTt programme (Hilsenbeck et al., 2016; Rieger et al., 2009). Thereby, an individual cell clone was investigated for as many division rounds as possible within the 96 h recording period. With the growth of the progeny, some daughter cells were lost during tracking, as reflected by question marks in the lineage trees (Fig. 2B-E). Using this strategy, we could compare cell cycle lengths of the WT and the KO in dependence of cytokines, irrespective of the size of the fraction of mitotic cells in the total NPC population that was clearly reduced in the KO. Phase contrast images revealed that after 96 h *in vitro* FGF2 treated cells had a phase bright, rounded cell body with two to three slender cell processes [Fig. 2A, Movie 3 (WT FGF2)]. In contrast, EGF treated cells displayed a less accentuated, somewhat larger cell body [Fig. 2A, see Movie 2 (WT EGF)]. Typical lineage trees of WT and Tnc knockout (KO) spinal cord progenitors showed an impressive synchronous cell cycle, generating morphologically similar sibling cells in all different conditions [Fig. 2B-E, see Movie 4 (Tnc KO EGF) and Movie 5 (Tnc KO FGF2) in the Supplementary Information]. While it was not possible to follow the further fate of individual daughters in our assay, the synchrony of division and the highly resembling phenotypes of resulting cells suggest that the majority of divisions at that stage appeared symmetric. Note, that the number of branching points of progenitors lacking Tnc is reduced in both EGF and FGF2 conditions compared to WT. This is in line with the observation of an overall reduced number of cell divisions in the mutant.

For the purpose to analyse the influence of the mitogens EGF and FGF2 on WT and Tnc KO spinal cord progenitors of the different generations, the cell cycle lengths were compared. The quantification of the data is summarized in Table 1. FGF2 treatment led to a faster cell cycle in the second, third, and fourth generations of WT spinal cord cells in comparison to EGF. A similar effect could be shown in the Tnc-deficient spinal cord progenitors. In the second and third generations the progenitors divided approximately 2 h faster each. Taken together the mitogen FGF2 boost the spinal cord progenitors to proliferate more quickly.

**Table 1.**
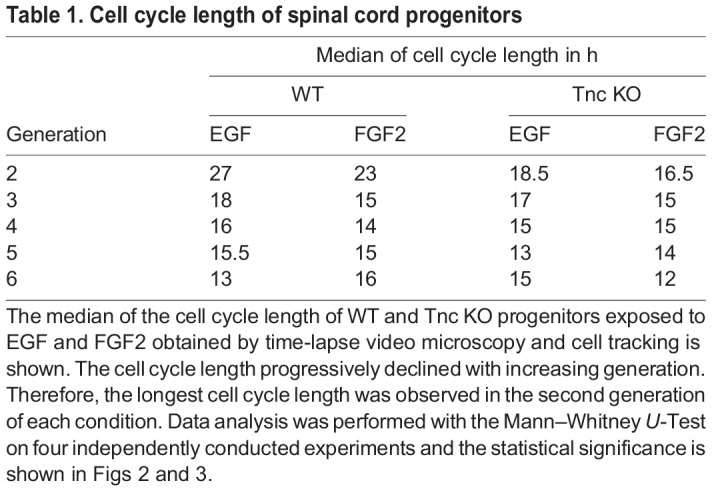
**Cell cycle length of spinal cord progenitors**

### The cell cycle length of progenitors adapts after 2 days *in vitro*

**Fig. 3.**
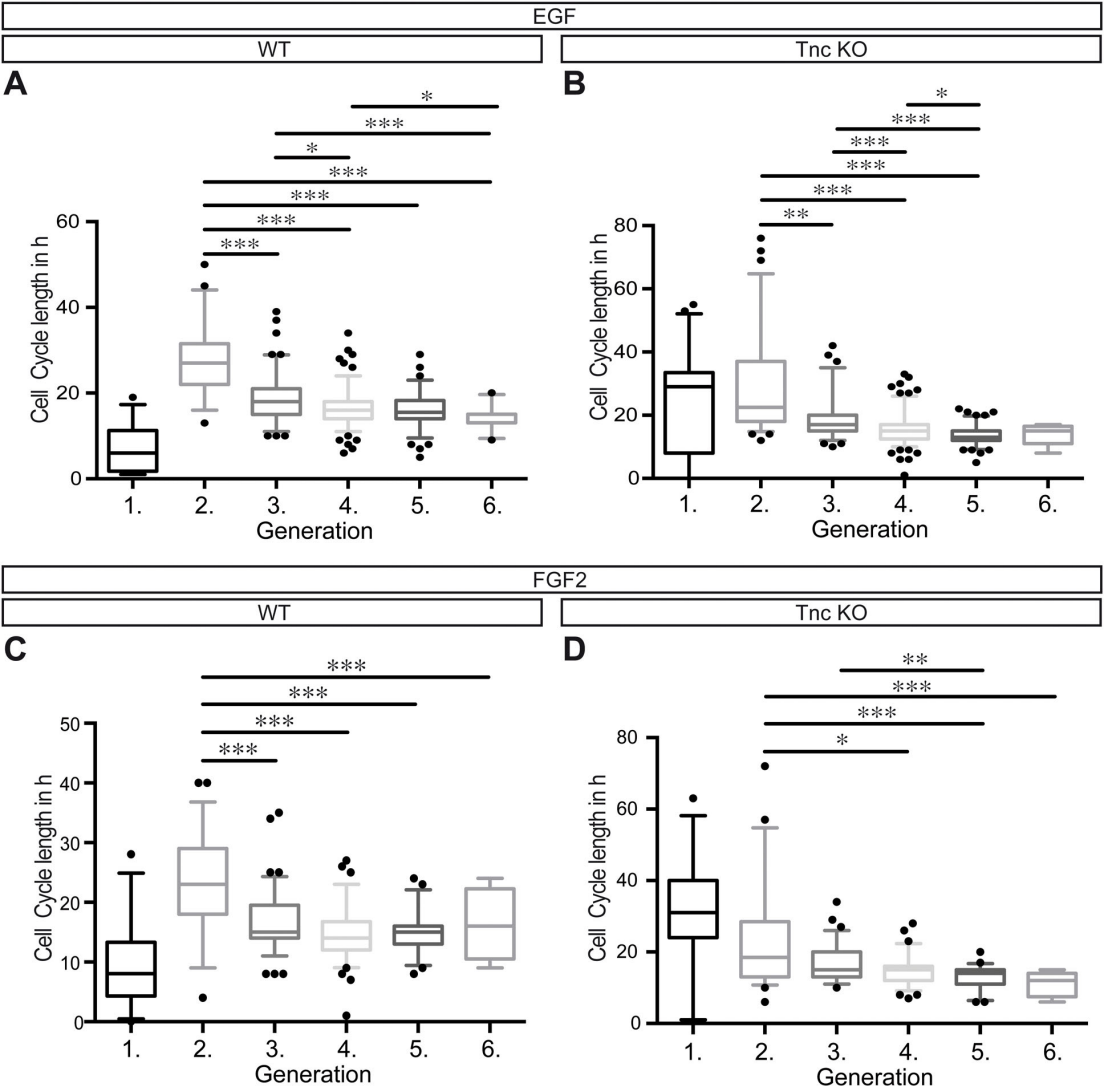
**WT and Tnc KO spinal cord progenitors divide faster with rising cell divisions.** Cell cycle length obtained by time-lapse video microscopy of WT (A,C) and Tnc KO (B,D) progenitors treated with either EGF (A,B) or FGF2 (C,D). (A) There is a strong decrease in the cell cycle length of WT progenitors from the second generation onwards, as well as from the third generation to the fourth, fifth and sixth generations. A similar result could be shown for Tnc KO progenitors (B). (A-D) Black dots indicate individual cells beyond the 5%-95% range of the box whisker plot. The FGF2 treatment both in WT (C) and Tnc KO (D) cells revealed that there is a reduction in the cell cycle length with ascending generations. *P≤0.05, **P≤0.01, ***P≤0.001 (Kruskal-Wallis Test with Dunn′s multiple comparisons test); *n*=4; percentile; 5%–95%.

**Fig. 4.**
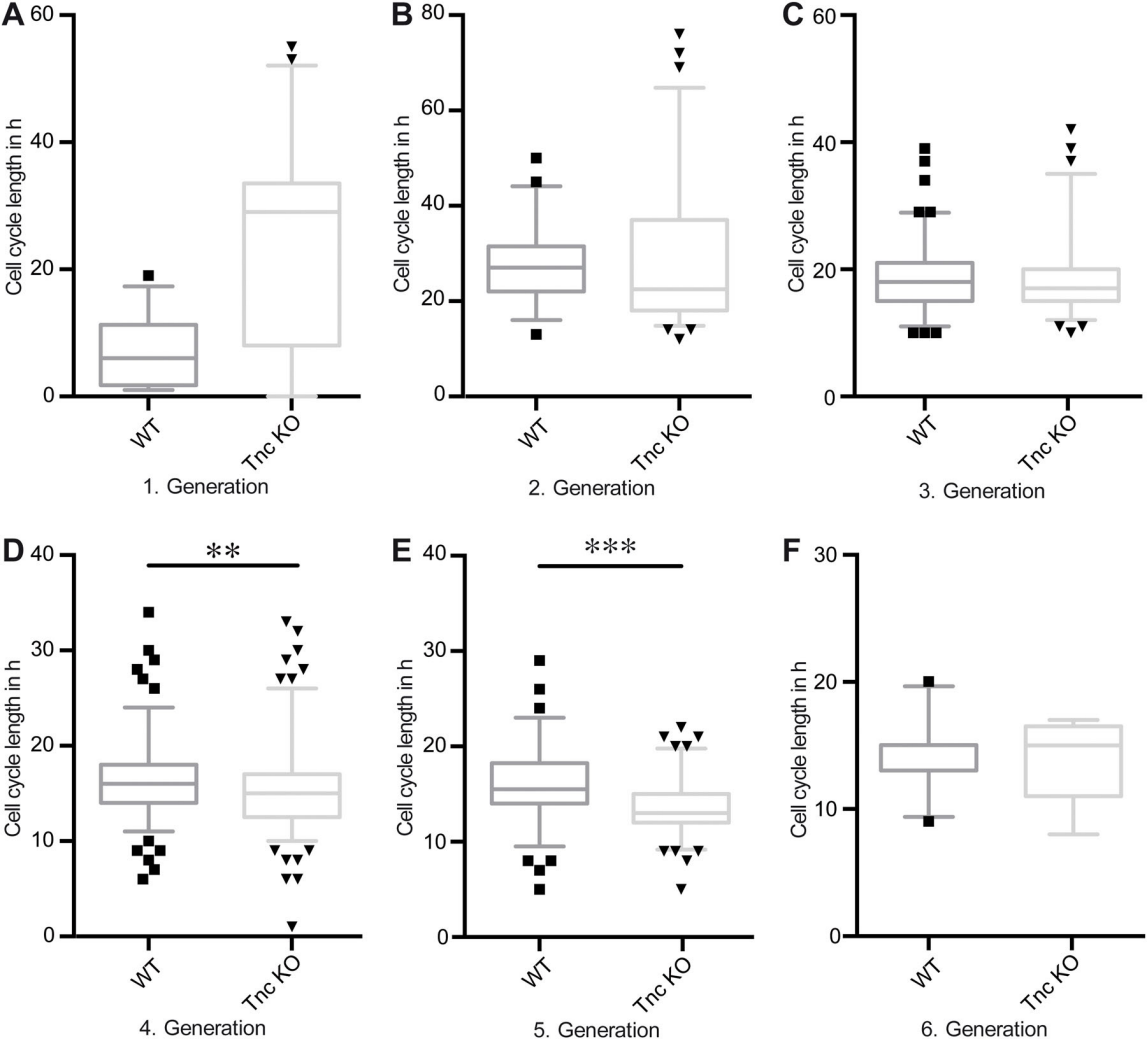
**Tnc deficiency leads to a faster cell cycle in the presence of EGF.** (A-F) Comparison between the cell cycle lengths of WT versus Tnc KO spinal cord progenitors treated with EGF within the different generations. Tnc KO progenitors divided much faster compared to WT progenitors. The difference was particularly visible in the fourth and fifth generations. Black squares indicate individual wild type and black triangles indicate individual Tnc KO cells beyond the 5%-95% range of the box whisker plot. *P*≤0.01, ***P≤0.001 (Mann-Whitney *U*-test); *n*=4; percentile: 5%–95%.

To get more information concerning the cell cycle length of WT and Tnc KO progenitors during the 4 days of recording, we compared the different generations with each other. Note that the first generation is not included in the analysis, because the state of the cell cycle of the firstly tracked progenitors could not be reliably defined. Nevertheless, the first cell division of WT progenitors occurred much faster in comparison with the Tnc KO progenitors. In all conditions it was obvious that the cell cycle length decreased and assimilated with rising generation (Fig. 3A-D and Table 1). The effect of mitogen application on WT progenitors was much stronger than on Tnc KO progenitors concerning the cell cycle length of the second generation compared to the third generation (Fig. 3A,C) (EGF: WT second generation, 27 h; third generation, 18 h. FGF2: WT second generation, 23 h; third generation, 15 h). The reduction of Tnc-deficient progenitors of the cell cycle length from generation two to three amounted to 1.5 h in both EGF and FGF2 condition (Fig. 3B,D). Taken together, after several rounds of division the cell cycle of the spinal cord progenitors appeared synchronized, but Tnc-deficient progenitors initially started off with a much slower cell cycle and were less responsive to EGF or FGF2 treatment than their WT counterparts (Fig. 4A).

### Tnc-deficient spinal cord progenitors divide faster upon EGF treatment

To directly compare the influence of Tnc on the cell cycle of WT with Tnc KO progenitors in the presence of EGF signalling we matched the corresponding data sets presented in Figs 2 and 3. The assessment of the cell cycle lengths of WT and Tnc KO progenitors indicated an overall tendency of Tnc deficient progenitors to divide faster. This effect was strongest in generation four and five. (Fig. 4D,E; fourth generation: WT, 16 h versus Tnc KO, 15 h; fifth generation: WT, 15.5 h versus Tnc KO, 13 h). We also considered the cell cycle length in an FGF2 dependent manner and we could detect analogous results between WT and Tnc KO progenitors as observed with the EGF treatment (Fig. S1A-F). Thus, Tnc exerts an influence of the cell cycle in response to EGF signalling.

### Embryonic spinal cord cells sustained their progenitor-like phenotypes in the presence of EGF and FGF

**Fig. 5.**
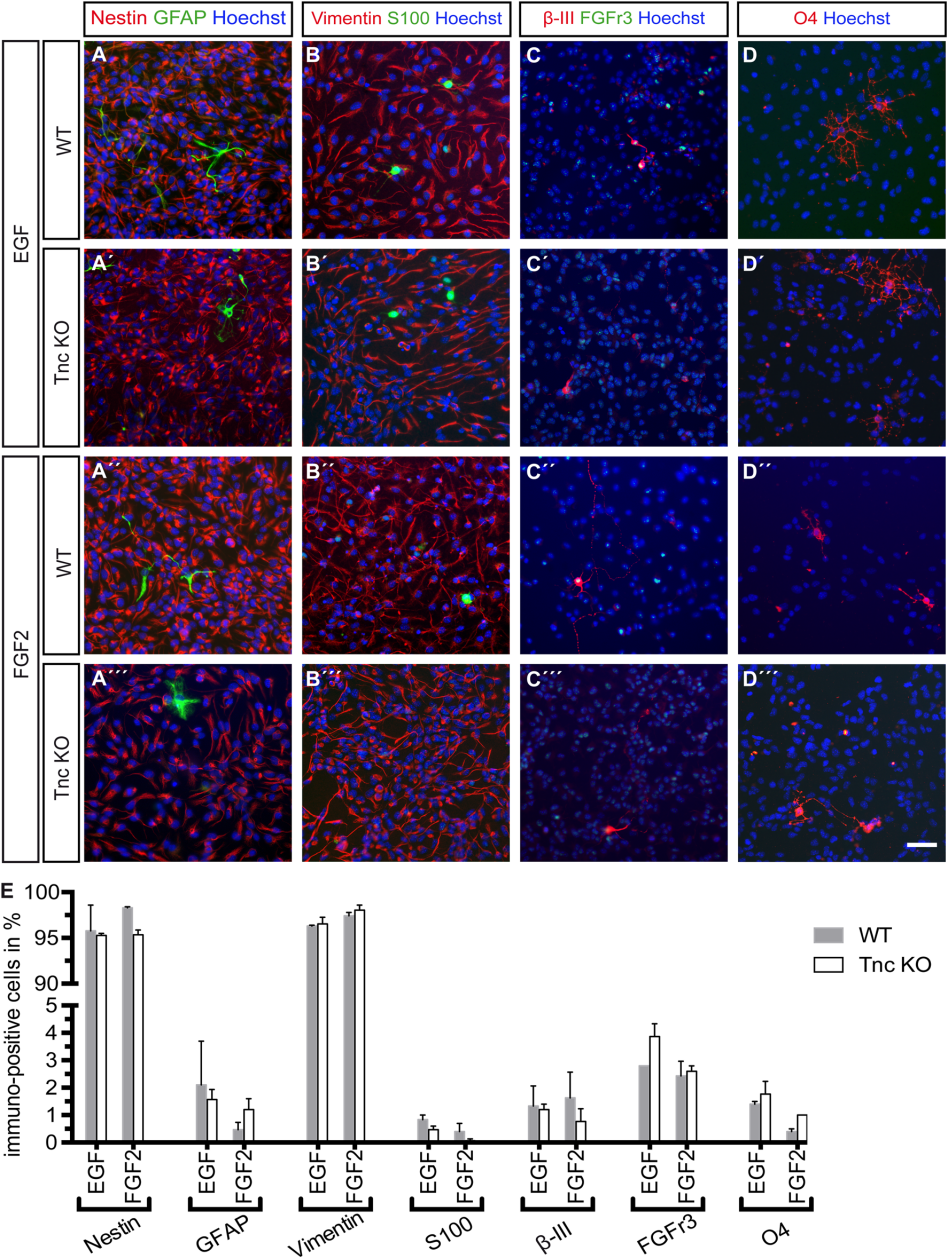
**The vast majority of WT and Tnc KO cells remained progenitors in the presence of EGF or FGF2.** (A-D‴) Immunocytochemical profile of progenitors kept continuously in the presence of cytokines under proliferating conditions. (E) Quantification showed that most cells were nestin- (A-A‴) and vimentin-positive (B-B‴) progenitors. Few immature and mature astrocytes (S100-positive, FGFr3-positive or GFAP-positive), Neurons (β-III-positive) and Oligodendrocytes (O4-positive) were detected. *n*=3; scale bar: 50 µm.

**Fig. 6.**
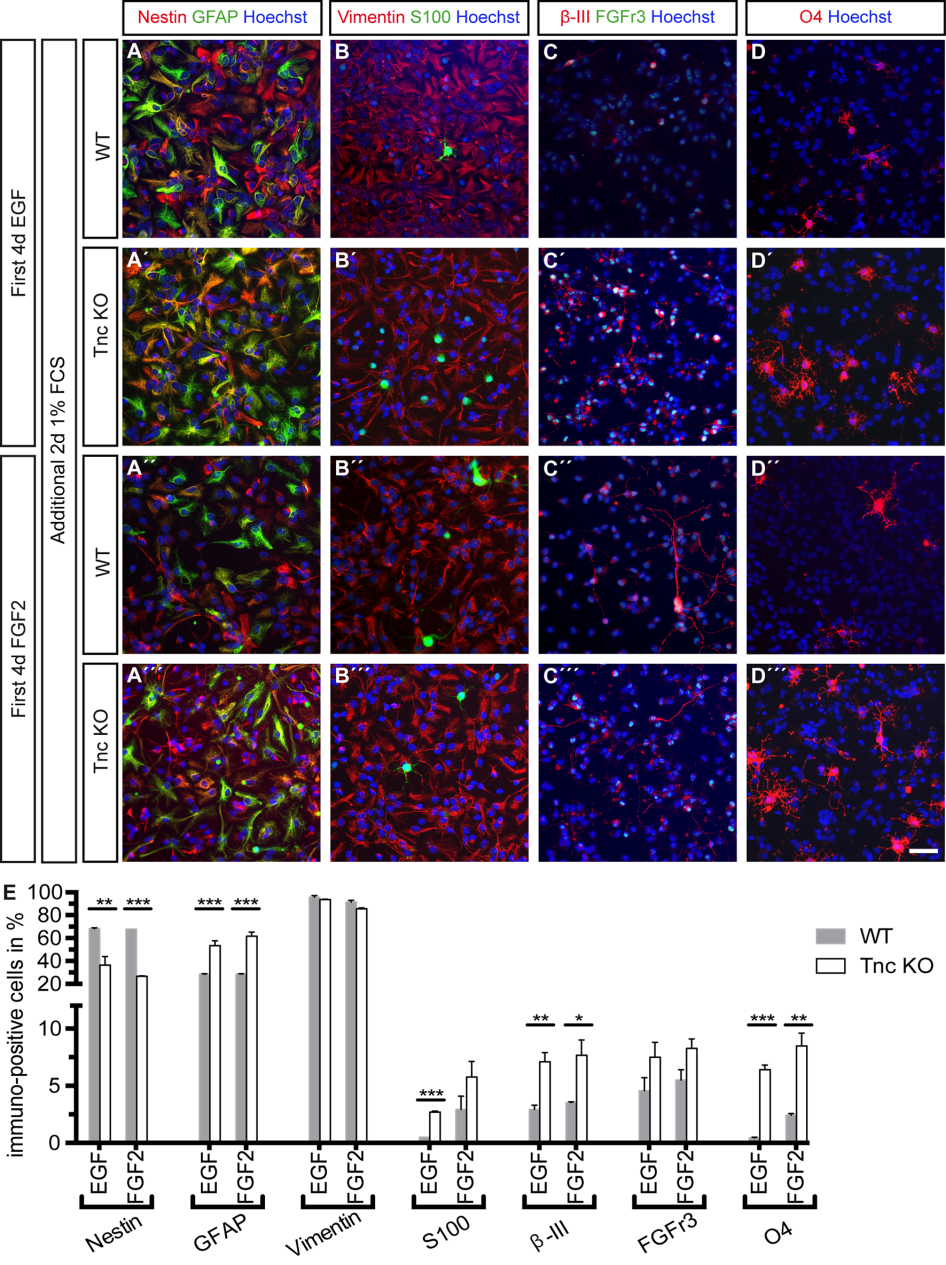
**Tnc deficiency resulted in an accelerated differentiation of spinal cord progenitors.** (A-D‴) In the first step, WT and Tnc KO progenitors were maintained in EGF or FGF2 under proliferative conditions. Thereafter, the cytokines were removed and the cultures were allowed to differentiate for two further days in the presence of 1% FCS. Immunocytochemical characterization using the indicated markers is illustrated. (E) The Tnc KO cells showed a more differentiated phenotype compared to WT cells and quantification revealed that more astrocytes (GFAP-positive, S100-positive or FGFr3-positive), Neurons (β-III-positive) and Oligodendrocytes (O4-positive) were generated, at the expense of progenitors (Nestin-positive). Error bars indicate s.d., *P≤0.05, **P≤0.01, ***P≤0.001 (*t*-test); *n*=3; scale bar: 50 µm.

Most of the Tnc expressing cells in the spinal cord at E15.5 are nestin-, vimentin- and GLAST-positive progenitors *in vivo* and we determined the cell identity of the progenitors at the end of the time-lapse video microscopy period (Karus et al., 2011). To this end, we performed a profiling of the WT and Tnc KO cells generated in presence of EGF and FGF2 *in vitro*. The average numbers of the quantification are presented in Table 2, which summarizes the column graphs (Figs 5E and 6E). The vast majority of cells remained nestin- and vimentin-positive progenitors (see Table 2 and Fig. 5A-B‴,E) and only few cells differentiated into immature FGFr3-positive and mature GFAP-positive astrocytes (see Table 2 and Fig. 5A-A‴,C-C‴), β-III-positive neurons (see Table 2 and Fig. 5C-C‴) and O4-positive oligodendrocytes (see Table 2 and Fig. 5D-D‴).

**Table 2.**
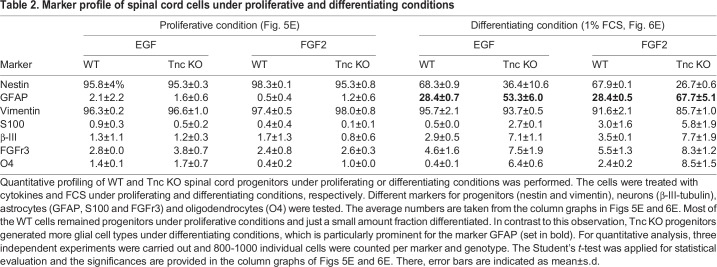
**Marker profile of spinal cord cells under proliferative and differentiating conditions**

The switch from FGF2- to EGF-responsiveness of NPCs is linked to the transition of neurogenesis to gliogenesis (Lillien and Raphael, 2000). For this reason we wanted to collect information regarding the capacity of EGF or FGF2 to drive spinal cord progenitors into distinct cell lineages. We differentiated spinal cord progenitors that had been pre-treated with the two mitogens for a further 2 days after withdrawal of the growth factors. There was an increase in GFAP-positive astrocytes at the expense of mostly nestin-positive progenitors in the Tnc KO cells in comparison with WT cells, independently of a prior exposure to EGF or FGF2 (see Table 2 and Fig. 6A-A‴). In addition to that, we observed more β-III-positive neurons, O4-positive oligodendrocytes and a little more S100-positive and FGFr3-positive immature astrocytes in Tnc KO compared to WT cells (see Table 2 and Fig. 6B-D‴). Based on this result, we propose that Tnc inhibits spinal cord progenitor differentiation and thereby contributes to maintenance of the stem cell compartment.

## DISCUSSION

**Fig. 7.**
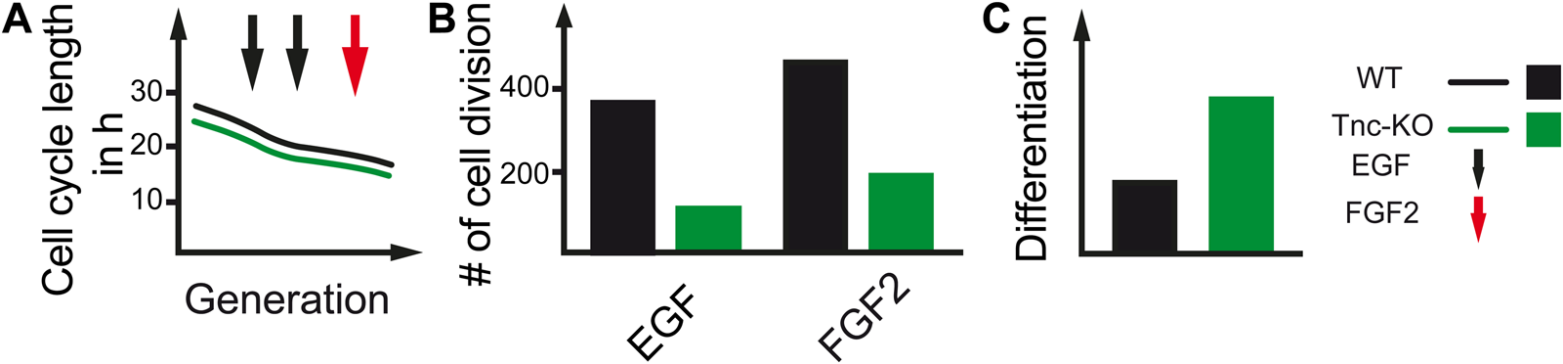
**Influence of Tnc on cell cycle length, cell division and differentiation.** (A) The cell cycle length is reduced with ongoing generation in both WT and Tnc KO spinal cord progenitors. This effect is much stronger in Tnc deficient progenitors in comparison with WT cells in an EGF-dependent manner. (B) Independently of the treatment with either EGF or FGF2, progenitors lacking Tnc divided less compared to WT progenitors. (C) Most likely, this led to a faster cell cycle exit, resulting in more differentiated cells and less progenitors when Tnc is absent.

Tnc is known for playing a crucial role in the embryonic and adult stem cell niches in the CNS of rodents and humans (Kazanis et al., 2007; Chiquet-Ehrismann et al., 2014; Pollen et al., 2015; Faissner et al., 2017). Previously we have shown that Tnc is expressed during spinal cord development and lack of Tnc altered FGF2 signalling, resulting in a delay of astrocyte maturation (Karus et al., 2011). Nevertheless, the influence of the ECM and in particular of the glycoprotein Tnc on the cell cycle is poorly understood. Here we show that FGF2 treatment could lead to a faster cell cycle in both WT and Tnc KO spinal cord progenitors in comparison with EGF (Fig. 7A). Furthermore, the overall proliferation of Tnc-deficient cells is reduced in response to FGF2 and EGF (Fig. 7B). Along these lines, we documented more differentiated cells in the absence of Tnc *in vitro*. In particular*,* the amount of GFAP-positive, S100-positive and FGFr3-positive astrocytes was increased at the expense of nestin-positive progenitors (Fig. 7C).

An increase of BrdU incorporation has been described in the E12 cortex and the E15 spinal cord of Tnc deficient mice (Garcion et al., 2004; Karus et al., 2011). While this observation suggests an impact on cell proliferation, not much is known about the relationship between Tnc and the cell cycle in spinal cord and cortical progenitors. However, there is evidence for the connection of Tnc and cell division in individual modified NIH 3T3 fibroblast, because a relationship between Tnc promoter up-regulation during the last 40% of the cell cycle and cell division could be detected (Halter et al., 2011). Previously we have performed microarray analysis of E15 WT and Tnc KO mouse spinal cords (Karus et al., 2011). We screened those data sets for genes, which are involved in processes like the cell cycle and cell proliferation. Indeed, we found an up-regulation of specific genes related to cell proliferation and the cell cycle summarized in Fig. S2A,B.

Early embryonic neural stem cells are FGF2-responsive and the accumulation of FGF2 fosters the expression of the EGF receptor at later developmental stages (Reynolds and Weiss, 1992; Lillien and Raphael, 2000). Due to the fact that the tracked spinal cord progenitors were cultivated as neurospheres prior to time-lapse video microscopy and retain nestin-positive and vimentin-positive progenitors (Fig. 6A-B‴,E), those cells might be rather in a self-renewing than differentiating mode. Therefore, the shorter FGF2-related cell cycle length (Fig. 2D,E) of both WT and Tnc KO progenitors in comparison with EGF exposure can be explained. The treatment with EGF and FGF2 of adult subependymal cells and cell tracking analysis showed similar results regarding a highly synchronous behaviour (Costa et al., 2011).

FGF signalling predominantly activates the MAPK signalling pathway (Corson et al., 2003) and cell proliferation occurs through the induction of cyclin D1 expression (Dailey et al., 2005; Ho and Dowdy, 2002). A faster cell cycle caused by shortening the G1 phase can be obtained by overexpression of the cell cycle genes cyclin D1, cyclin E1 or Cdk4/cyclin D1 (Lange et al., 2009; Nonaka-Kinoshita et al., 2013) and sustains the proliferation of progenitors. Tnc could act as a modulator of cell cycle progression and we observed that a loss of Tnc leads to a shorter cell cycle length in the presence of EGF. The EGF-like repeats of Tnc can act as a low affinity binding partner for the EGF receptor and provoke stimulation of the MAPK signalling pathway resulting in proliferation of NR6 fibroblasts (Iyer et al., 2007; Swindle et al., 2001). Infusion of EGF into the lateral ventricle of the adult rat and mouse brains massively increases SVZ progenitors proliferation (Doetsch et al., 2002; Kuhn et al., 1997). Interestingly, we observed an overall lower number of cell divisions in Tnc KO progenitors upon exposure to both EGF and FGF2 in comparison to the wild type. This probably led to a faster cell cycle exit of at least a subpopulation of progenitors, resulting in more differentiated cells and less progenitors when Tnc is absent. In E15.5 Tnc KO spinal cord tissue, the expression of the EGF-receptor is reduced around the ventral central canal, where Tnc is strongly upregulated in WT animals at this developmental time point. In the Tnc KO spinal cord, the emergence of EGF responsiveness is delayed (Karus et al., 2011). Along the same lines, Tnc regulates the expression of the EGF-receptor in telencephalic progenitors (Garcion et al., 2004). Although the cell cycle length in Tnc-deficient progenitors is less affected with respect to FGF2-dependent signalling, there is evidence for a direct binding of FGF2 to the fifth FNIII domain of Tnc (De Laporte et al., 2013).

In general, the formation of the ECM in the stem cell niche of mouse and human differs, which reflects the lissencephalic and gyrencephalic appearance of the neocortex. Transcriptome analysis of subpopulations of mouse neural precursor cells (NPCs) according to their specific germ layers’ identities illustrated that ECM components in the SVZ are reduced compared to the VZ (Fietz et al., 2012). In the human brain, the VZ, the inner SVZ and the outer SVZ display a specific ECM composition, which might modulate the proliferative capacity of human basal progenitors (Fietz et al., 2012). Furthermore, a recent study has shown that Tnc is highly expressed in the human outer SVZ, where a particular population of basal progenitor stem cells resides (Pollen et al., 2015). In the same vein, an overexpression of Pax6, a marker of radial glia and a well-known regulator of Tnc in the mouse brain (von Holst et al., 2007) increases the number of proliferating basal progenitors in the mouse brain, expanding this cell pool otherwise characteristic for the primate cortex (Asami et al., 2011; Wong et al., 2015). These findings, in conjunction with our observations focusing on spinal cord progenitors, underline the potential impact of Tnc on the cell cycle and the proliferation of spinal cord progenitors.

Under differentiating conditions, we documented more glial cells in the absence of Tnc (see scheme in Fig. 7). This effect of Tnc on the glial lineage has been shown *in vivo* where more immature astrocytes were transiently generated (Karus et al., 2011). Oligodendrocyte precursor cell differentiation is inhibited by Tnc as well (Czopka et al., 2010, 2009; Garwood et al., 2004). Interestingly, Tnc is needed for normal proliferation and morphology of astrocytes in primary cultures (Ikeshima-Kataoka et al., 2007).

In conclusion, the cell cycle length of spinal cord progenitors lacking Tnc is faster in the presence of both the cytokines EGF and FGF2 in comparison with WT cells. Tnc deficiency results in less proliferation. Under differentiating conditions, Tnc KO progenitors display a differentiation bias towards glial cells. Based on these findings, we suggest that Tnc supports and modulates the process of progenitor proliferation, presumably by acting on the cell cycle in conjunction with cytokines.

## MATERIALS AND METHODS

### Animals

Embryos (E15) of time mated pregnant WT and *Tnc* homozygous knockout mutants (Forsberg et al., 1996), both in the 129sv background, were used for the experiments. The age of the embryos was identified according to the Theiler Stages and the day of the vaginal plug was defined as embryonic day (E) 0.5.

### Neurosphere culture

The neurosphere culture system has been described previously (Karus et al., 2011). Briefly, the lumbosacral spinal cord of E15-old embryo was isolated, incubated with 30 U/ml Papain (Worthington, New Jersey, USA) and the dissociated cells were grown in neurosphere medium consisting of DMEM/F-12 (1:1), 0.2 mg/ml L-glutamine (all from Sigma-Aldrich), 2% (v/v) B27, 100 U/ml penicillin, 100 μg/ml streptomycin (all from Invitrogen), 20 ng/ml FGF2, 20 ng/ml EGF (both tebu-bio, Offenbach, Germany) and 0.25 U/ml heparin (Sigma-Aldrich) for 6-7 days to get neurospheres (von Holst et al., 2006).

### Cell culture of time-lapse video microscopy, proliferation and differentiation assay

The neurospheres were centrifuged for 5 min at 80 ***g*** and the cell pellets were enzymatically digested with 0.05% trypsin-EDTA in HBSS (Invitrogen) for 25-30 min at 37°C to obtain a single-cell suspension. The digestion was stopped with 1 ml ovomucoid [1 mg/ml trypsin inhibitor (Sigma-Aldrich), 50 μg/ml BSA and 40 μg/ml DNaseI (Worthington, New Jersey, USA) in L-15 medium (Sigma-Aldrich)] and after the mechanical dissociation the single-cell suspension was centrifuged for 5 min at 80 ***g***. The cells were re-suspended in neurosphere medium. 24-well plates (Thermo Fisher Scientific) for time-lapse video microscopy and four-well dishes (Greiner, Kremsmünster, Austria) for the proliferation assay were sequentially coated with 10 µg/ml (w/v) poly-D-lysin (Sigma-Aldrich) in ddH_2_O, followed by 10 μg/ml laminin-1 (Invitrogen) in PBS for 1 h at 37°C each. In order to perform time-lapse video microscopy the cells were plated at a density of 30,000 cells/well. For the proliferation assay 10,000 cells/well were seeded in proliferation medium consisting of neurosphere medium containing either 20 ng/ml EGF or 20 ng/ml FGF2 with 0.25 U/ml heparin and incubated at 37°C and 6% (v/v) CO_2_. After 4 days with regard to the proliferation assay the cells were immunocytochemically stained and some cells were further cultivated for 2 days at 37°C and 6% (v/v) CO_2_ after the proliferation medium was replaced by differentiation medium consisting of neurosphere medium containing 1% (v/v) FCS.

### Immunological reagents

The following primary antibodies were used in this study: the monoclonal antibodies were: anti-βIII tubulin (1:500: mouse IgG, clone SDL3D10; Sigma-Aldrich), anti-FGFr3 (1:150: rabbit IgG; Santa Cruz Biotechnology), anti-GFAP (1:150: mouse IgG, clone GA5; Sigma-Aldrich), anti-nestin (1:500: mouse IgG; Millipore), anti-O4 (1:30; mouse IgM) (Sommer and Schachner, 1981), anti-vimentin (1:300: mouse IgG, clone LN-6; Sigma-Aldrich). The polyclonal antibodies were: anti-GFAP (1:300: rabbit IgG; Dako, Hamburg, Germany), anti-S100 (1:300: rabbit IgG; Dako). The specific secondary antibodies used in this study were CY2- (1:300) or CY3-coupled (1:500) anti-mouse and anti-rabbit antibodies (all from Dianova, Hamburg, Germany).

### Immuncytochemistry

The immuncytochemical staining was performed according to an established protocol (von Holst et al., 2006). Briefly, after removal of the culture medium, the adherent cells were washed twice with KRH/A consisting of 125 mM NaCl, 4.8 mM KCL, 1.3 mM CaCl_2_×2H_2_O, 1.2 mM MgSO_4_×7 H_2_O, 1.2 mM KH_2_PO_4_, 5.6 mM D-Glucose, 25 mM HEPES, 0.1% (w/v) bovine serum albumin (BSA), pH 7.3 and in order to detect the membrane-bound O4 epitope, the cells were incubated for 20 min with the O4 antibody diluted in KRH/A. Then the cells were washed with KRH and fixed for 15 min with 4% (w/v) PFA in PBS (137 mM NaCl, 3 mM KCl, 6.5 mM Na_2_HPO_4_×2H_2_O, 1.5 mM KH_2_PO_4_ pH 7.3). Afterwards all cells were washed with PBT1 (PBS with 1% (w/v) BSA, 0.1% (w/v) Triton X-100, pH 7.3) twice and incubated with all other primary antibodies diluted in PBT1 against intracellular epitopes for at least 30 min. After washing the cells twice with PBS/A [PBS with 0.1% (w/v) BSA, pH 7.3], the cells were incubated with species-specific fluorochrome-labelled secondary antibodies to detect the different primary antibodies and Bisbenzimid (1:10^5^) to visualize the nuclei (all diluted in PBS/A). Before the cells were mounted in PBS/glycerine (1:1), all cells were washed with PBS. All steps were performed at room temperature.

### Time-lapse video microscopy and microscopy

The time-lapse microscopy of spinal cord progenitors was conducted at the Axiovert 200M with the AxioCam HRm camera and a self-written VBA module remote (Rieger and Schroeder, 2009) controlling the Zeiss Axiovision program 4.8.1 (all Zeiss, Jena, Germany). Additionally, the devices Tempcontrol 37-2 digital and CTI-Controller 3700 digital (both Zeiss) were used to create defined culture conditions with 37°C and 6% (v/v) CO_2_. Phase contrast images were taken every 5 min for at least 96 h. Single-cell tracking was performed using tTt, a computer program (Hilsenbeck et al., 2016; Rieger and Schroeder, 2009). Movies were created using ImageJ 1.45r (National Institutes of Health) software and are played at a speed of five frames per second. The immunofluorescence pictures were taken with the Axioplan2 microscope equipped with the AxioCam MRm using Axiovision 4.8.2 software (all from Zeiss).

### Data analysis

To analyse the time-lapse video microscopy data, the Kruskal-Wallis Test with Dunn's multiple comparisons test or the Mann–Whitney *U*-Test were used. Four independent experiments for each mouse genotype were performed. The number of tracked cells is listed in Tables S1 and S2. The data are illustrated as box whisker plots with percentiles from 5% to 95%. To quantify the proliferation and differentiation assays, 800-1000 individual cells per three independent experiments were used and antibodies were counted for each mouse genotype. The Student's *t*-test was used to analyse the data. Error bars are indicated as mean±s.d. All statistics and graphs were performed using Graphpad Prism^®^ 6 software (GraphPad Inc.). *P*-values are given as *P≤0.05, **P≤0.1 and ***P≤0.001.

## Supplementary Material

Supplementary information
