# Supplementary material for: Cell tracking in vitro reveals that the extracellular matrix glycoprotein Tenascin-C modulates cell cycle length and differentiation in neural stem/progenitor cells of the developing mouse spinal cord
Source: Biol Open. 2018 Jul 15;7(7):bio027730. doi: 10.1242/bio.027730 (PMC6078350; doi:10.1242/bio.027730)
Supplement: Supplementary information [file biolopen-7-027730-s1.pdf]

Supplementary data

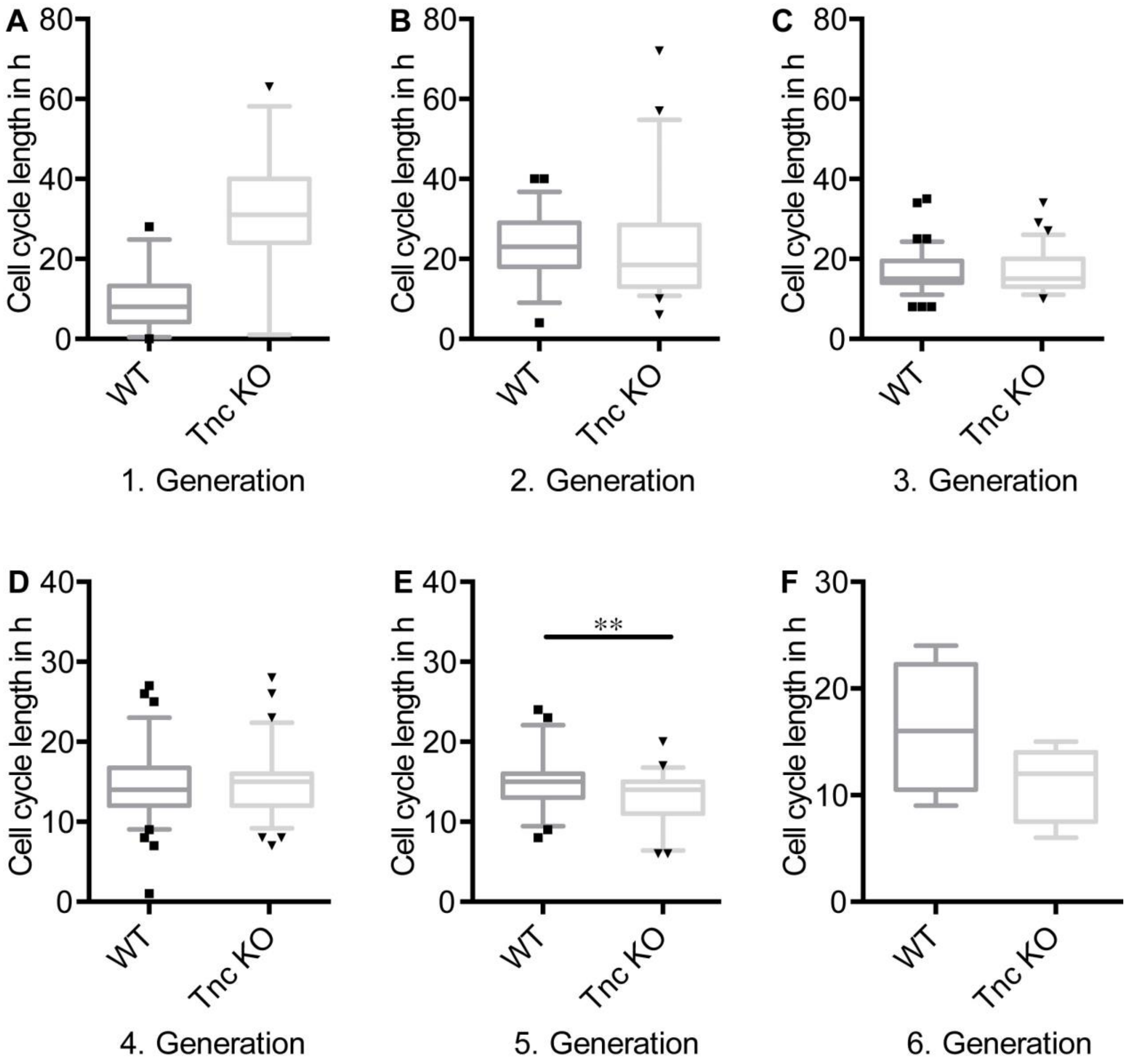

Figure S1

FGF2 treatment did not change the cell cycle length Tnc KO of spinal cord progenitors. (A-F) The cell cycle length of WT and Tnc KO cells in the presence of FGF2 is compared obtained by cell tracking. The time period between divisions of Tnc deficient progenitors is conflictive with regard to the various generations. There was a significant decrease in generation 5 (E), but just slight decreases in generation 2. (B) and 6. (F), whereas the opposite effect can be seen in generation 4. Mann-Whitney U-test; n=4; Percentile: 5%-95%

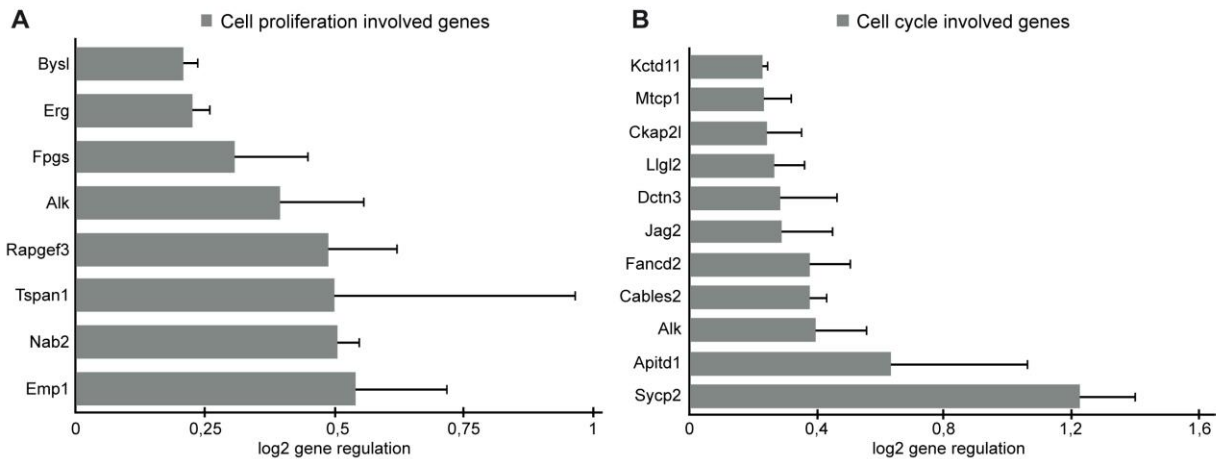

Figure S2

The loss of Tnc leads to an upregulation of specific genes *in vivo*. We compared WT with Tnc KO E15 spinal cord tissue via microarray analysis (for detailed information see Karus et. al 2011) and found genes involved in cell cycle to be upregulated. Those genes were Synaptonemal complex protein 2 (SYCP2), apoptosis-inducing, TAF9-like domain 1 (Apitd1), anaplastic lymphoma kinase (Alk), CDK5 and Abl enzyme substrate 2 (Cables2), Fanconi anemia, complementation group D2 (Fancd2), jagged 2 (Jag2), dynactin 3 (Dctn3), lethal giant larvae homolog 2 (Llgl2), cytoskeleton associated protein 2 like (Ckap2) and mature T-cell proliferation 1 (Mtcp1). Furthermore, genes related to the process of cell proliferation like epithelial membrane protein 1 (Emp1), NGFI-A binding protein 2 (Nab2), tetraspanin 1 (Tspan1), Rap guanine nucleotide exchange factor 3 (Rapgef3), anaplastic lymphoma kinase (Alk), folylpolyglutamate synthase (Fpgs), ETS transcription factor (Erg) and bystin like (Bysl) were upregulated.

Biology Open • Supplementary information

Table S1

Total number of cells per genotype tracked in the presence of EGF

Generation	1		2		3		4		5		6	
Genotype	WT	Tnc KO	WT	Tnc KO	WT	Tnc KO	WT	Tnc KO	WT	Tnc KO	WT	Tnc KO
Total # of tracked cells	40	40	57	76	100	98	140	169	94	103	26	5

Legend: Four independent experiments for each genotype, WT and Tnc KO, were conducted. Cell tracking of initial 10 “mother-cells” and all of their sibling-cells was performed over a period of 96h for every single experiment. That is why 40 cells were tracked in the 1. generation of both genotypes. With ongoing generations the number of tracked cells differs, because cells weren´t trackable anymore or sometimes the cells died.

Table S2

Total number of cells per genotype tracked in the presence of FGF2

Generation	1		2		3		4		5		6	
Genotype	WT	Tnc KO	WT	Tnc KO	WT	Tnc KO	WT	Tnc KO	WT	Tnc KO	WT	Tnc KO
Total # of tracked cells	40	40	55	54	93	78	80	71	48	43	4	5

Legend: Four independent experiments for each genotype, WT and Tnc KO, were conducted. Cell tracking of initial 10 “mother-cells” and all of their sibling-cells was performed over a period of 96h for every single experiment. That is why 40 cells were tracked in the 1. generation of both genotypes. With ongoing generations the number of tracked cells differs, because cells weren´t trackable anymore or sometimes the cells died.

Biology Open • Supplementary information

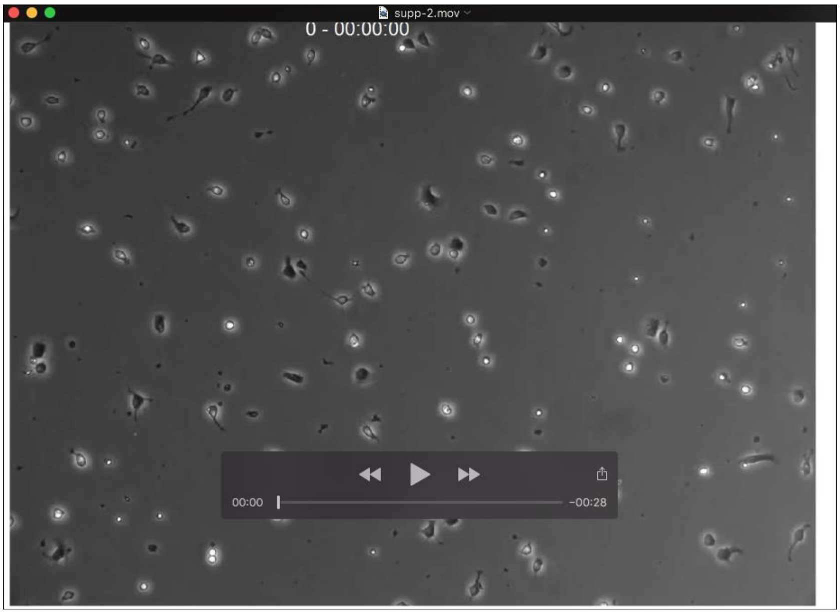

Movie 1

Spinal cord progenitors divided rarely without any growth factors. The spinal cord progenitors divided just once or twice during the whole 4 day period of time-lapse video microscopy. Many cells died and the surviving cells displayed different types of morphologies.

Biology Open • Supplementary information

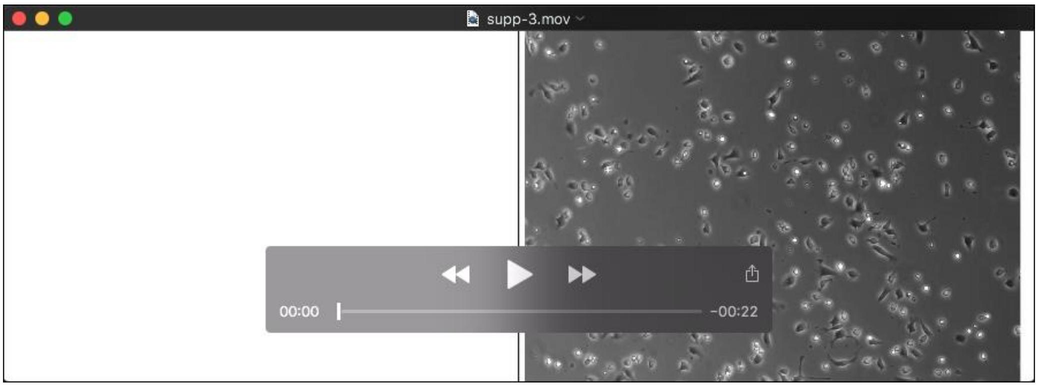

Movie 2: **Lineage tree of tracked WT spinal cord progenitors exposed to EGF.**

NPCs obtained from E15 spinal cords were cultivated in the presence of either EGF or FGF2 and followed over a period of 96 hours. In the movies you can follow simultaneously the creation of the lineage tree (left side) of a specific cell (right side) and all of their siblings marked with red numbers obtained by time-lapse video microscopy. Tracking was performed with the programme tTt.

Biology Open • Supplementary information

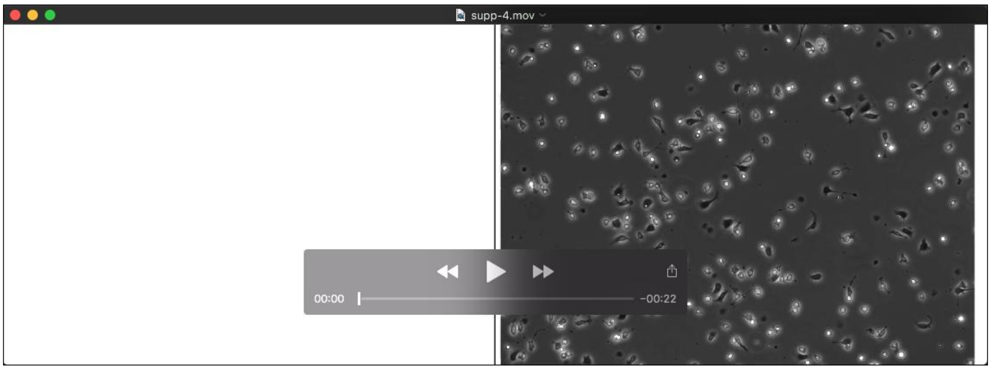

Movie 3: **Lineage tree of tracked WT spinal cord progenitors exposed to FGF2.** NPCs obtained from E15 spinal cords were cultivated in the presence of either EGF or FGF2 and followed over a period of 96 hours. In the movies you can follow simultaneously the creation of the lineage tree (left side) of a specific cell (right side) and all of their siblings marked with red numbers obtained by time-lapse video microscopy. Tracking was performed with the programme tTt.

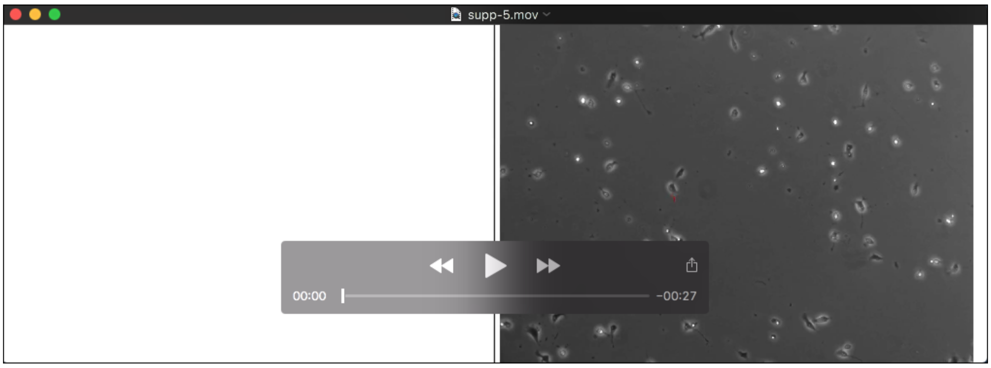

Movie 4: **Lineage tree of tracked Tnc KO spinal cord progenitors exposed to EGF.** NPCs obtained from E15 spinal cords were cultivated in the presence of either EGF or FGF2 and followed over a period of 96 hours. In the movies you can follow simultaneously the creation of the lineage tree (left side) of a specific cell (right side) and all of their siblings marked with red numbers obtained by time-lapse video microscopy. Tracking was performed with the programme tTt.

Biology Open • Supplementary information

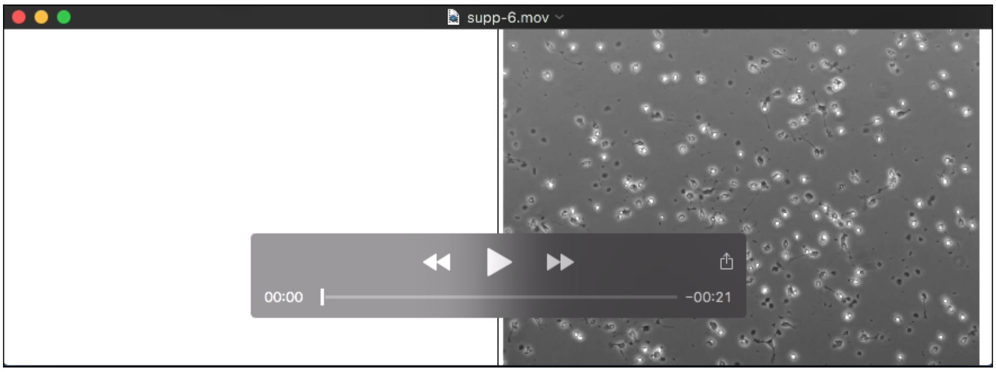

Movie 5: **Lineage tree of tracked Tnc KO spinal cord progenitors exposed to FGF2.**

NPCs obtained from E15 spinal cords were cultivated in the presence of either EGF or FGF2 and followed over a period of 96 hours. In the movies you can follow simultaneously the creation of the lineage tree (left side) of a specific cell (right side) and all of their siblings marked with red numbers obtained by time-lapse video microscopy. Tracking was performed with the programme tTt.

Biology Open • Supplementary information
